# Mental health consequences of military sexual trauma: results from a national survey in the French military

**DOI:** 10.1186/s12889-022-12545-x

**Published:** 2022-02-02

**Authors:** Caroline Moreau, Sandrine Duron, Dina Bedretdinova, Aline Bohet, Henri Panjo, Nathalie Bajos, Jean Baptiste Meynard

**Affiliations:** 1 Soins et Santé Primaire, CESP Centre for research in Epidemiology and Population Health, U1018, Inserm, F-94807 Villejuif, France; 2 Department of Population, Family and Reproductive Health, Johns Hopkins Bloomberg School of Public Health, 615 North Wolfe Street, Baltimore, MD 21205 USA; 3 French Military Center for Epidemiology and Public Health, Marseille, France; 4 INSERM, UMR S912, « Economic & Social Sciences for Health and Processing of Medical Information » (SESSTIM), F-13385 Marseille, France; 5 IRIS Interdisciplinary Research Institute on Social Issues Social Sciences Politics Health, U997 Inserm – EHESS, F-93322 Aubervilliers, France; 6 French Military Medical Academy, Ecole du Val-de-Grâce, Paris, France

**Keywords:** Sexual assault, Sexual harassment, Depression, Posttraumatic stress disorder, Military, Gender disparities, France

## Abstract

**Background:**

Military sexual trauma (MST) is a major public health concern, given its prevalence and mental health sequelae. This phenomenon is particularly prevalent among women in the US military, although more cases involve men given their overrepresentation. Little is known about MST and its consequences in other military settings, including in Europe.

**Methods:**

This study draws from a national survey in the French military, including 1268 servicemen and 232 servicewomen. We conducted bivariate and multivariate analysis, using simple and multinomial logistic regressions to evaluate the associations between different forms of MST (repeated sexual comments alone/one form of sexual oppression (coercion, repeated verbal unwanted attention or assault)/ several sexual stressors) and symptoms of depression and of positive post-traumatic stress disorder (PTSD) screening scores.

**Results:**

Women were both more likely to experience MST and to experience more severe forms of MST than men. Women were also more likely than men to report mental health symptoms (31% *versus* 18% for symptoms of depression and 4.0% *versus* 1.8% for positive PTSD screening scores). Different forms of MST were associated with different levels of psychological distress. Women reporting repeated sexual comments alone had higher odds of depressive symptoms (OR=3.1 [1.7, 5.5]) relative to women with no MST. Likewise, the odds of depressive symptoms were 6.5 times higher among women and 8.0 times higher among men who experienced several sexual stressors relative to those who reported no MST. We also found higher relative risk of subthreshold PTSD screening scores among women reporting any form of sexual stressor, including sexual comments alone (RRR = 4.5 [2.8, 7.4]) and an elevenfold increase in the relative risk of positive PTSD screen scores (RRR = 11.3 [2.3, 55.6]) among women who experienced several sexual stressors relative to women with no MST.

**Conclusion:**

MST is associated with mental health distress among service members in the French military, especially for women. The heightened risk of MST coupled with psychological sequelae call for preventive programs to reduce MST and for screening programs to provide adequate psychological support.

**Supplementary Information:**

The online version contains supplementary material available at 10.1186/s12889-022-12545-x.

## Background

Military sexual trauma (MST) is defined by the US federal law as a psychological trauma resulting from sexual violation while serving on active duty and is increasingly recognized as a major public health concern, given its prevalence and health sequelae [1–5]. MST involves a range of sexual violations along a spectrum of harm, from unwelcomed sexual advances, requests for sexual favours and other verbal, behavioural and or physical conduct of a sexual nature (generally referred to as sexual harassment [SH]), as well as attempted or forced sexual contact, characterized as sexual assault (SA) [6]. A meta-analysis based on 69 articles conducted in the US military estimates that on average 13.9% of veterans and service members have experienced sexual assault and 31.2% have experienced sexual harassment during their service in the military [3]. Data in non-US settings are scarce, but recent analysis in the French military suggests significant exposure with one in three women and 15% of men reporting recent SH and 9% of women and 3.3% of men experiencing SA in the last 12 months [7]. These figures, based on the COSEMIL study (Comportements Sexuels chez les Militaires), a multi-thematic national study on sexual health in the French military [8] were the first MST estimates in the French military. They not only represent alarming human rights violations but have profound implications for service people’s health and wellbeing, especially as MST may have more damaging health consequences for military personnel as compared to civilians [1]. In particular, military personnel are at a heightened risk of re-victimization given their living arrangements and they face multiple barriers to accessing support, due to deployment, fear of social ostracism and sanctions related to social hierarchy, group cohesion and gender stereotypes [1, 4, 9]

A substantial body of work conducted among US military populations substantiates an association between MST and mental health disorders, including major depressive symptoms, substance abuse, PTSD symptoms and suicidal ideation [1, 4, 10]. While estimates vary across studies, mental health distress is consistently associated with MST across military populations, including males and females [11, 12], veterans and active duty members, and deployed and non-deployed personnel [5, 10]. The investigation of psychological response to a continuum of sexual aggression generally indicates stronger associations in the case of SA compared to other forms of sexual stressors [13, 14]. Few studies acknowledge the cumulative effects of sexual stressors on mental health, despite high correlations between SA and SH, especially in the military context [13, 15]. Street and colleagues acknowledge this phenomenon in a study among US reserve forces, reporting the odds of depression associated with SH in the absence of SA were in the range of 1.75 to 2.21 for male and female reservists but rose to 4.41 and 5.38 respectively when associated with SA [13]. Other authors have described more severe and prolonged forms of mental illness in the presence of multiple experiences of sexual trauma [1].

Most research on the sequelae of MST focus on women, who are more likely than men to be exposed to MST, including its more severe forms [12]. A few authors who have explored gender patterns of mental health disorders related to MST suggest more deleterious effects for men than women, as men may be more likely to experience repeated physical assault, are less likely to disclose and seek care and are less likely to receive social support than women as sexual aggression threatens their gender identity [10]. Other studies, however, have reported greater psychological distress following sexual harassment among women [12].

The previous findings mostly pertain to the US context; there is limited investigation of MST health sequelae in other military environments, with only one study from South Korea reporting a relationship between MST and psychological distress [16]. The present study aims to expand our understanding of the mental health sequalae of MST, by evaluating the associations between a spectrum of sexual stressors and mental health in the French Military context, which operates under different social norms and presents different working conditions and policies than the US military. We first explored how different forms of MST are related to depressive symptoms and to a positive PTSD screening score, after adjusting for individual and workplace characteristics. We also examined how patterns of sexual stressors combined to inform mental health symptoms. Finally, we investigated how the aforementioned associations differed between men and women.

## Methods

We used data from the COSEMIL study, the first national sexual health survey in the French military, conducted in 2014-2015 [8]. The survey included a two-stage random sampling strategy. Initially, 18 military units were randomly selected after stratification by location (Mainland France, or overseas bases) and military branch (Army, Navy, Air force), with unequal probabilities of selection based on unit size. Next, 120 individuals per unit were randomly selected with oversampling of women (1 woman for 5 men, as women account for only 16% of the French military workforce). Selected individuals were invited by the unit commander to participate in an information session during on-duty hours and subsequently provided informed consent to participate. All participants were informed about the goals, procedures and voluntary nature of the study before providing consent to participate. Participation was anonymous, and participants incurred no sanctions if they declined participation. Altogether, 1268 men and 232 women aged 18-57 years were included. The participation rate was 76% (14% did not attend the study information session, 9% refused participation and 1% were excluded due to software deficiency during data collection). The COSEMIL study was approved by the French government oversight agency (Commission Nationale Informatique et Liberté, N° 2014-100) and the Comité de Protections des Personnes Sud Méditarranéen II (N° 2013-AOOO47-38).

Participants were asked to complete a 37-minute questionnaire on laptops, providing information on a range of topics including sociodemographic characteristics, sexual attitudes and behaviours as well as sexual health outcomes: sexually transmitted infections (STI), sexual violence, unintended pregnancy, and sexual dysfunction. The questionnaire also investigated other health-related concerns including mental health.

In this study, we considered two outcome measures: depressive symptoms and a positive screen for PTSD. Depressive symptoms were assessed using the CESD-10 instrument and dichotomized into two categories (presence or absence of symptoms of depression) using a threshold of 10, as recommended by Radloff [17]. The PTSD screen score was derived from the PTSD Checklist-Military questionnaire (PCL-M), corresponding to Diagnostic and Statistical Manual of Mental Disorders IV classification [18]. We created a three category variable based on the total symptom severity PCL-M score: no positive PTSD screen (scores <34), subthreshold PTSD screen (scores 35 to 49) and PTSD positive screen (score =>50). The subthreshold category of PTSD screen score was used given its frequency (16.5% based on self-reports in US military populations) and associations with other psychopathologies [19]. We checked that our severity score measure was consistent with a symptom cluster definition of PTSD, based on DSM-IV criteria, including one re-experiencing symptom, three numbing/avoidance symptoms and two hyperarousal symptoms [18]. We found that 97% of PTSD scores =>50 were classified as a positive PTSD screen based on cluster symptom criterion and therefore only report on the total severity score measure.

Our key independent factors related to MST in the last 12 months. Measures were derived from three sources presented in Table 1: the DoD-SEQ instrument [20], the French national survey on violence (Virage) [21], and the 2006 national sexual survey conducted in France [22]. We defined several composite measures based on four forms of aggression occurring in the military context in the last 12 months: receipt of repeated sexual comments, unwanted verbal sexual attention, sexual coercion, and sexual assault (unwanted touching, attempted rape or forced sex). We created a dichotomous measure of sexual oppression (SO), which included more personal and severe forms of aggression [23]: sexual coercion, repeated verbal unwanted sexual attention or sexual assault. We also created a four categorical measure capturing a graduation of MST severity and a cumulation of experiences: 1) no MST 2) MST in the form of repeated sexual comments alone, 3) MST involving 1 type of sexual oppression (either coercion or repeated verbal unwanted sexual attention or sexual assault), 4) MST involving several types of sexual stressors including at least one form of sexual oppression.

**Table 1:** Measures and distribution of sexual aggression in the form of sexual harassment or assault in the workplace in the last 12 months

	In the last 12 months in the context of your work (responses options no/once, more than once)	Source	Men % (n)	Women % (n)
**Repeated sexual comments**	**Any repeated sexual comments (any of the following situations experienced more than once)**		14.6% (159)	30.3% (67)
	You heard sexist comments or jokes that made you uncomfortable?	DoD		
	You were put ill at ease by pictures of a sexual nature	DoD		
	Someone has had sexual remarks or attitudes that have put you ill-at-ease – ex: questions about private life, salacious remarks, looks that undress, mimes of sexual gestures?	DoD		
**Repeated unwanted sexual attention**	Someone insistently made you sexual proposals, despite your refusal?	DoD	0.9% (13)	8.4% (19)
**Sexual coercion (any of the following situations)**			0.4% (4)	4.9% (10)
	You were suggested a reward or special treatment if you had sexual relations	DoD		
	You felt threatened if you were not sexually cooperative,	Virage		
	You were treated badly because you refused sexual relations You felt threatened if you were not sexually cooperative,	DoD DoD		
**Sexual assault (any of the following situations)**	Someone touched your breasts, your buttocks, squeezed you, cornered you to kiss you, rubbed themselves against you against your will?	Virage	2.9% (43)	7.8% (18)
	Attempted or forced sex in the last 12 months	CSF	0.5% (6)	1.7% (4)
	Total		*3.3% (47)*	*9.0% (20)*
**Sexual oppression**	**(repeated unwanted verbal attention, coercion or assault)**		4.2% (59)	14.9% (43)

A number of covariates representing individual attributes (age, level of education, financial situation, army rank), partnership attributes (partnership situation and same sex partnerships) and work environment factors (deployment in the last 12 months, army type, unit-level sex ratio, gender attitudes and collegiality) associated with MST [7] were considered. Group level measures of collegiality and gender attitudes were created by averaging individual-level responses within each unit, after excluding the participant’s own response.

We examined the prevalence of symptoms of depression and PTSD positive screen according to respondents’ sociodemographic characteristics. We then evaluated bivariate and multivariate associations between three indicators of MST (harassment, assault and oppression) and mental health outcomes (symptoms of depression and PTSD). Finally, we conducted multivariate logistic regression models to assess the association between a four categorical measure of sexual aggression (1 - none, 2- SH in the form of repeated sexual comments alone, 3- SH involving coercion, repeated unwanted sexual attention or assault) and symptoms of depression, adjusting for covariates. A multinomial regression model was used to assess the association between sexual aggression and PTSD screen, defined in three categories (no PTSD, sub threshold screen for PTSD, positive screen for PTSD). We also conducted sensitivity analysis using the PTSD symptom cluster definition to assess the robustness of our results based on PTSD screening definition. All analyses were stratified by sex to identify sex-differences in the associations between sexual aggression on mental health. We also tested for sex differences in the association between sexual stressors and psychological distress in a fully adjusted model including both sexes. Analyses were conducted in Stata 14.2 and were weighted to account for the complex survey design as well as non-participation (post-stratification weights were applied to match the distribution of sex, age and army rank in the French military).

## Results

The sample description is displayed in Table 2. The mean age of participants was 33.1 years for men and 30.6 years for women. Most respondents were cohabitating with a partner and more than half of men and 37% of women had children. Women completed more education than men and were less likely to be in difficult financial situation. Conversely, men were more likely to be officers than women.

**Table 2:** Characteristics of French active-duty servicemen and women included in the COSEMIL

	Gender	Men	Women	Total
		% (N)	% (N)	% (N)
	Total	100.0 (1268)	100.0 (232)	100.0 (1500)
**Age in 3 groups**	<25	19.1 (239)	17.8 (55)	19.0 (294)
	25-29	23.6 (264)	36.6 (68)	25.1 (332)
	>=30	57.3 (765)	45.6 (109)	55.9 (874)
**Cohabitation with current partner**	Everyday	58.0 (768)	58.8 (124)	58.1 (892)
	Not every day	24.4 (283)	23.1 (64)	24.3 (347)
	No current partner	17.6 (216)	18.1 (43)	17.7 (259)
**Children**	No	45.2 (561)	62.5 (140)	47.2 (701)
	Yes	54.8 (705)	37.5 (92)	52.8 (797)
**Marital status**	Married	38.7 (540)	25.3 (61)	37.1 (601)
	Civil partnership	11.9 (142)	20.7 (37)	12.9 (179)
	Single	45.3 (525)	48.1 (120)	45.6 (645)
	Divorced	4.0 (51)	5.9 (13)	4.3 (64)
	Widow(er)	0.1 (2)	0.0 (0)	0.1 (2)
**Same sex partnerships**	No	97.1 (1233)	84.6 (200)	95.7 (1433)
	Yes	1.7 (17)	15.4 (32)	3.3 (49)
	Never had sex	1.2 (18)	0.0 (0)	1.1 (18)
**Place of birth**	Mainland France	86.3 (1099)	88.6 (202)	86.6 (1301)
	Overseas France	10.1 (120)	8.0 (23)	9.9 (143)
	Foreign country	3.6 (49)	3.4 (7)	3.6 (56)
**Level of education**	Less than High school	42.8 (542)	30.0 (69)	41.3 (611)
	High school graduation	34.5 (468)	46.1 (114)	35.9 (582)
	>High school	22.7 (256)	23.9 (48)	22.9 (304)
**Financial situation**	No problem	43.6 (589)	48.3 (122)	44.1 (711)
	Difficult/Just enough	56.4 (674)	51.7 (109)	55.9 (783)
**Alcohol**	Non drinker/Non risky drinker	61.4 (731)	66.0 (141)	61.9 (872)
	Risky drinker	38.6 (512)	34.0 (86)	38.1 (598)
**Military branch**	Army	61.7 (580)	39.6 (73)	59.2 (653)
	Air force	18.3 (381)	43.6 (97)	21.2 (478)
	Navy	20.0 (307)	16.8 (62)	19.6 (369)
**Army rank**	Enlisted personnel	43.3 (562)	46.9 (119)	43.7 (681)
	NC-Officer	44.6 (587)	46.8 (99)	44.9 (686)
	Officer	12.1 (119)	6.3 (14)	11.4 (133)
**Type of contract**	Career	37.8 (480)	21.9 (48)	36.0 (528)
	Temporary Contract	62.2 (787)	78.1 (184)	64.0 (971)
**Deployment in the last 12 months**	No	65.5 (892)	87.3 (193)	68.0 (1085)
	Yes	34.5 (376)	12.7 (39)	32.0 (415)
**Sexual aggression in the last 12 months**	No sexual aggression	82.5 (1068)	64.3 (153)	80.4 (1221)
	MST in the form of repeated sexual comments alone	13.3 (141)	20.9 (44)	14.2 (185)
	MST involving 1 form of sexual oppression (coercion/ unwanted attention/ or assault)	2.8 (38)	4.6 (11)	3.0 (49)
	MST involving multiple sexual stressors	1.4 (21)	10.3 (24)	2.4 (45)

Sexual stressors in the last 12 months were common, with 35.8% of women and 17.5% of men reporting any MST, mostly in the form of repeated sexual comments alone (20.9% of women and 13.3% of men) (Table 2). Coercion or unwanted verbal sexual attention were less common (10.8% of women and 1.1% of men) and 9.0% of women and 3.3% of men reported a sexual assault in the last 12 months (Table 1). Altogether, 4.2% of men and 14.9% of women had an experience qualifying as sexual oppression (coercion, unwanted attention, or assault) (Table 1) and 1.4% of men and 10.3% of women experienced multiple sexual stressors (Table 2)

Almost a third of women (31%) and 18% of men presented with CESD depressive symptom scores of 10 or more, meeting the criteria for symptoms of depression (Table 3). Having a positive screen for PTSD based on total severity scores was less common, affecting 4.0% of women and 1.8% of men, while 15.3% of women and 7.1% of men presented with subthreshold PTSD screen scores (Table 3).

**Table 3:** Proportion of servicemen and servicewomen in the French military reporting depressive symptoms (CESD >=10) and having a positive PTSD screening score (sub-threshold PTSD screening score: PCLS = 35-49; positive PTSD screening score: PCLS = >50) according to sociodemographic characteristics and MST experiences

			Men			Women	
		% depressive symptoms	% Sub threshold PTSD screening score	% Positive screening score for PTSD	% depressive symptoms	% Sub threshold PTSD screening score	% Positive screening score for PTSD
Total		18.2%	7.1%	1.8%	31.3%	15.3%	4.0%
Age	25 years	17.4	5.8	1.9	50.8	17.3	4.4
	25-29 years	15.1	7.6	1.3	27.3	8.6	0.6
	30+ years	19.7	7.4	2.0	27.0	19.9	6.5
Current partner	Cohabitating all the time	14.8	6.2	1.6	21.9	12.5	2.9
	Not every day	20.2	6.9	2 .1	46.4	23.8	8.5
	No current partner	26.6	10.5	1.9	42.7	13.7	1.6
Same sex partnership	Yes	25.3	16.1	0.0	35.2	19.9	0.7
	No	18.3	7.0	1.8	30.6	14.5	3.4
	Never had sex	2.3	0.0	0.0			
Children	Yes	18.6	7.4	1.6	22.1	2.6	0.5
	No	17.8	6.8	2.1	11.2	4.7	6.8
Place of birth	Mainland France	17.7	6.8	1.6	16.2	4.5	3.6
	Overseas France/Foreign country	20.9					0.0
Education level	<High school	19.7	7.9	2.3	19.8	2.8	0.9
	High school graduation	18.1	5.8	1.9	14.3	2.0	0.8
	>High school	15.0	7.8	0.6	12.0	9.1	8.0
Financial situation	No problem	10.9	5.6	0.9	11.1	2.4	3.9
	Tight or Difficult	24.0	8.3	2.5	19.3	5.3	2.4
Military branch	Army	19.5	6.6	2.3	14.2	2.0	0.7
	Air force	15.9	8.8	1.6	20.6	7.3	10.1
	Navy	16.3	7.0	0.3	4.1	0.0	0.0
Army rank	Officer	20.1	2.0	0.0	7.8	14.2	2.2
	Junior Officer	17.9	6.9	1.7	15.8	3.4	0.8
	Enlisted personnel	12.4	8.7	2.4	15.8	3.1	12.9
Deployment in the last 12 months	Yes	17.0	4.0	2.1	15.1	12.3	2.2
	No	18.8	8.8	1.6	15.3	2.7	3.7
No MST in the last 12 months		17.5	7.1	1.4	21.9	10.5	1.3
MST in the form of repeated sexual comments alone		17.6	5.2	3.2	38.6	24.1	3.7
MST including 1 form of sexual oppression (coercion or repeated unwanted or Sexual Assault)		18.4	10.6	3.1	50.8	22.2	7.5
MST including several sexual stressors		62.8	16.8	5.9	66.5	24.5	19.1

Symptoms of depression and positive PTSD screen scores were elevated among service-members reporting MST (Table 3). Thus, the proportion of symptoms of depression was higher among women who experienced repeated sexual comments alone, as well as women who experienced one form of sexual oppression (coercion, repeated verbal unwanted attention or assault) or multiple sexual stressors compared to those who reported no MST (*p* = 0.002). Similar patterns were noted for men exposed to multiple sexual stressors while symptoms of depression were not substantially increased among those who experienced only one form of sexual stressor (Table 3). Subthreshold and positive screening for PTSD were more common among women who experienced any form of MST, whether comments alone, sexual oppression or multiple forms of sexual stressors compared to those who experienced no MST in the last 12 months (*p* = 0.01). A greater proportion of servicemen who experienced sexual oppression had subthreshold levels of PTSD screening scores or a positive screen score for PTSD compared to those who reported no MST (*p* = 0.01).

Associations between sexual aggression and mental health were mostly confirmed in multivariate analysis. We found that women reporting any form of MST (repeated sexual comments alone or sexual oppression) had elevated odds of experiencing symptoms of depression relative to those with no MST. The odds of symptoms of depression were 6 times higher among women who reported multiple sexual stressors relative to those who reported no MST (*p*<0.001). Men who experienced multiple sexual stressors also had eight times the odds of experiencing symptoms of depression relative to those who reported no MST (*p* = 0.03) (Table 4). The associations between sexual comments alone and symptoms of depression (*p* = 0.04) and between one form of sexual oppression and symptoms of depression (*p* = 0.05) were greater for women than men while the effect of sexual oppression was similar for both sexes (*p* = 0.96). Other factors related to symptoms of depression included age, celibacy, being in a difficult financial situation (for men), workplace factors like sex ratio and collegiality (also for men) and being in the Air Force or Navy (for women) (Appendices 1 and 2).

**Table 4:** Odds of depressive symptoms and risks of a positive screen for PTSD according to experiences of MST in the last 12 months among servicemen and servicewomen in the French military: results from multivariate simple and multinomial logistic regressions including unit level clustering

	Men			Women		
	Depressive symptoms	subthreshold PTSD screening score	Positive PTSD screening score	Depressive symptoms	subthreshold PTSD screening score	Positive PTSD screening score
	aOR 95%CI	aRRR 95%CI	aRRR 95%CI	aOR 95%CI	aRRR 95%CI	aRRR 95%CI
No MST	Ref	Ref	Ref	Ref	Ref	
Sexual Harassment (repeated sexual comments, coercion or unwanted verbal attention)	1.3 [0.7,2.6]	1.2 [0.5, 3.0]	2.9 [0.7, 13.1]	3.7^**^ **[2.5, 5.5]**	3.5** [2.3, 5.3]	**-**
No MST	Ref	Ref	Ref	Ref	Ref	
Any Sexual assault (unwanted touching or attempted or forced sex)	1.9 [0.9,4.1]	0.6 [0.1, 3.0]	3.7 [0.5, 29.0]	**2**^**.**^6* [1.1,6.0]		
No MST	Ref	Ref	Ref	Ref	Ref	-
repeated sexual comments alone	1.0 [0.5,1.9]	0.7 [0.2, 2.6]	3.1 [0.6-17.1]	3.1^***^ **[1.7,5.5]**	4.5*** [2.89, 7.4]	
MST involving 1 form of sexual oppression (coercion/ repeated unwanted or SA)	0.9 [0.5,1.9]	1.1 [0.4, 3.2]	2.4 [0.2-27.9]	5.2^***^ **[1.9,13.9]**	3.9** [1.6, 9.6]	5.1 [0.3, 83.5]
MST involving several sexual stressors	8.0*[1.2-52.5]	4.0*[1.3, 12.2]	4.3 [0.6-33.7]	6.5^***^ **[2.6,16.0**]	3.0** [2.1, 4.2]	11.3**[2.3,55.6]

Model adjusted for age, cohabitation status, education, financial situation, sexual orientation, army rank, military branch, deployment in the last 12 months, military branch, low acceptance of increasing female representation in the army, low social cohesion, higher female representation

^*^ *p* < 0.05, ^**^ *p* < 0.01, ^***^ *p* < 0.001

Men and women who experienced any form of MST were more likely to have elevated PTSD based on total severity screening scores (*p*<0.001 for both sexes). Specifically, women who reported repeated sexual comments alone, sexual oppression or several sexual stressors had elevated relative risks of subthreshold PTSD screening scores. In addition, women’s risk of having a positive PTSD screening score was 11 times higher when they reported multiple sexual stressors compared to those with no MST, while men who reported multiple sexual stressors had four times the relative risk of having a subthreshold PTSD screening score (Table 4). Sensitivity analysis using the binary PTSD symptom cluster indicator showed that the odds of a PTSD positive screen were 3.27 (1.73-6.17) times higher among servicemen who reported multiple sexual aggressions and 16.34 (1.99-134.126) times higher among women who experienced sexual oppression. The association between sexual comments and a subthreshold PTSD screening score was marginally stronger for women than men (*p* = 0.06) while associations between other sexual stressors and PTSD scores were similar by sex. Age and having same sex partnerships were associated with having a positive screening score for PTSD among men (Appendix 1), while age, education military branch and army rank were related to positive PTSD screening scores for women (Appendix 2).

## Discussion

In line with previous studies conducted mostly in the US military [1, 2, 5, 10], MST was a common experience in the French military with substantial implications for mental health. While patterns of sexual aggression were similar for both sexes, most commonly involving repeated sexual comments or unwanted touching, the gender gap was profound, as women were twice as likely as men to report any MST and to experience the most severe forms of sexual stressors. These gender inequalities are pervasive across all settings, including in the general population in France, and in other military settings in the US [12], underscoring the need for gender-transformative interventions to tackle sexual aggression in the workplace.

Consistent with studies conducted in the US, servicemen and women exposed to MST were substantially more likely to express symptoms of depression and to have positive PTSD screening scores [1, 4]. Results also indicate different forms of aggression were associated with different levels of psychological distress mirroring the findings of a study conducted among army reservists in the United States [13], albeit distinguishing more specific events that are less severe, but more commonly and repeatedly experienced, such as sexual comments. These associations remained even after adjusting for a range of potential confounders, including individual as well as workforce characteristics, generally not accounted for in previous studies.

Our results support the continuum of harm framework [24, 25], endorsed by the US Department of Defense, that acknowledges all forms of aggressions rather than SA alone, in an effort to prevent SA. While the frequency of mental health sequelae was higher when forms of aggression were more severe, results also indicate that women exposed to repeated sexual comments alone had higher levels of depressive symptoms, which was not the case for men. Given the prevalence of these forms of sexual stressors, these results support the need to expand the scope of prevention programs to address verbal sexual stressors, not only as a point of prevention of SA, but also to address prevalent mental health conditions related to these forms of harassment. The benefit of these preventive measures expands beyond the sphere of mental health, as harassment and psychological conditions have sexual health implications, including on STI and sexual dysfunctions [26, 27].

Studies exploring sex-differences in psychological distress following sexual harassment report conflicting results, some suggesting no sex-differences [28, 29], while others describe stronger associations among women [30, 31], but don’t include a formal test of interaction. The present study provides evidence of gender disparities as associations between sexual stressors and psychological distress (depressive symptoms and subthreshold PTSD screening scores) were statistically stronger for women compared to men. Gender disparities in MST sequalae are likely due to differences in the nature of MST experiences, social support systems and access to healthcare [11], which were not assessed in the present study. Additional qualitative research is needed to better understand how men and women experience and cope with sexual stressors in the French military context to guide the institutional response. The need to address women’s psychological distress is pressing as they suffer the cumulative burden of being more likely to be exposed to MST, of experiencing more severe forms of MST and of suffering greater psychological sequela from these sexual stressors [11].

These results need to be interpreted with a number of limitations in mind. The MST metrics in the COSEMIL study used fewer items from the SEQ DoD [7], which were adapted to the cultural context [32] and allowed comparisons with the population-based Virage study in France [21]. Unlike the revised SEQ DoD [33], perceived severity of MST events was not investigated, although only repeated events were considered as SH. Likewise, the psychological measures used in this study are based on screening instruments which do not equate with diagnoses of mental health disorders. In addition to measurement concerns, the female sample size was small, despite oversampling of servicewomen, resulting in large confidence intervals. The cross-sectional nature of the study also prevents causal interpretation, although the study’s conclusions are mostly consistent with findings of retrospective cohort studies, in which sexual trauma precedes the onset of mental illness [34]. In addition, the cross-sectional design allows investigation of recent events (MST in the last 12 months) that are not available in retrospective administrative data, all while adjusting for workplace environment,. Finally, unobserved factors, such as other work-related traumatic events, race/ethnicity and childhood adverse events could confound or moderate the associations explored.

Despite these limitations, this study adds to the growing literature on the implications of MST on mental health outcomes in several ways. First, the multidimensional measures of sexual stressors show how common forms of sexism mostly neglected from SA prevention programs specifically affect women’s mental health, increasing the profound gender gap in mental health illness in military populations. Second, unlike most research on MST, the COSEMIL study includes a national probability sample of active-duty personnel, allowing an investigation of mental health sequalae following MST in a representative sample of service members operating in a range of military settings [8]. While a number of studies, mostly using convenience samples or administrative data have reported similar associations in the US military [6], there is little investigation of MST sequalae in other contexts, including Europe, deterring programmatic action to integrate systematic screening and treatment to reduce the health consequences of MST. The study results serve as an impetus for action beyond the US military setting.

## Conclusion

Experiences of MST are related to an elevated risk of poor psychological health in the French military, especially among women. The heightened risk of sexual aggression coupled with greater psychological sequelae calls for preventive policies to tackle MST, by challenging the gender order in the military, through normative and institutional change to promote gender equality. Such policies coupled with screening programs to provide adequate psychological support are needed to reduce SH and SA and their sequalae.

## Supplementary Information

**Additional file 1 : Appendix 1.** Odds of depressive symptoms and risks of positive PTSD score according to sexual stressors in the last 12 months among servicemen in the French Military: results from multivariate and multinomial logistic regressions including unit level clustering. **Appendix 2.** Odds of depressive symptoms and risks of positive PTSD score according to sexual stressors in the last 12 months among servicewomen in the French Military: results from multivariate and multinomial logistic regressions including unit level clustering.

## Data Availability

The COSEMIL dataset is not publicly available to protect confidentiality as the dataset contains indirectly identifiable information. A request for a de-identified subset of the COSEMIL data can sent to the general’s office at dcssa-essd-eeps.contact.fct@intradef.gouv.fr.
