# Supplementary material for: Mental health consequences of military sexual trauma: results from a national survey in the French military
Source: BMC Public Health. 2022 Feb 2;22:214. doi: 10.1186/s12889-022-12545-x (PMC8812201; doi:10.1186/s12889-022-12545-x)
Supplement: Supplementary file 1 — Additional file 1 : Appendix 1. Odds of depressive symptoms and risks of positive PTSD score according to sexual stressors in the last 12 months among servicemen in the French Military: results from multivariate and multinomial logistic regressions including unit level clustering. Appendix 2. Odds of depressive symptoms and risks of positive PTSD score according to sexual stressors in the last 12 months among servicewomen in the French Military: results from multivariate and multinomial logistic regressions including unit level clustering. [file 12889_2022_12545_MOESM1_ESM.docx]

Appendix 1. Odds of depressive symptoms and risks of positive PTSD score according to sexual stressors in the last 12 months among servicemen in the French Military: results from multivariate and multinomial logistic regressions including unit level clustering.

|  |  | Depressive symptoms CESD score >10 vs CESD<=10 | | | Subtheshold PTSD vs No PTSD | | | | Positive PTSD scorevs No PTSD | | | |  |
| --- | --- | --- | --- | --- | --- | --- | --- | --- | --- | --- | --- | --- | --- |
|  |  | aOR | 95%CI | | | aRRR | 95%CI | | | aRRR | 95%CI | | |
|  | No MST |  |  |  | |  |  |  | |  |  |  | |
| MST | SH in the form of repeated sexual comments alone | 1.0 | 0.5 | 2.0 | | 0.7 | 0.2 | 2.6 | | 3.1 | 0.6 | 17.1 | |
|  | 1 form of sexual oppression* | 0.9 | 0.5 | 1.9 | | 1.1 | 0.4 | 3.2 | | 2.4 | **0.2** | **27.9** | |
|  | Several sexual stressors | **8.0** | **1.2** | **52.5** | | **4.0** | **1.3** | **12.2** | | 4.3 | **0.6** | **33.7** | |
| Age | 18-24 |  |  |  | | Ref |  |  | |  |  |  | |
|  | 25-29 | 0.9 | 0.5 | 1.7 | | **2.0** | **1.3** | **3.1** | | 0.7 | 0.1 | 3.7 | |
|  | >=30 | **2.0** | **1.1** | **3.5** | | **3.0** | **1.7** | **5.4** | | 1.5 | 0.5 | 4.0 | |
| Cohabitation with current partner | Everyday |  |  |  | | Ref |  |  | |  |  |  | |
|  | Not every day | 1.7 | 0.9 | 3.0 | | 1.2 | 0.4 | 3.4 | | 1.3 | 0.4 | 5.0 | |
|  | No current partner | **2.6** | **1.7** | **4.1** | | 1.8 | 0.6 | 5.2 | | 1.4 | 0.1 | 8.6 | |
| Education level | <High school |  |  |  | | Ref |  |  | |  |  |  | |
|  | High school graduation | 1.2 | 0.7 | 1.9 | | 0.9 | 0.6 | 1.3 | | 1.0 | 0.3 | 3.7 | |
|  | >High school | 1.2 | 0.7 | 2.0 | | 1.8 | 0.6 | 5.6 | | 0.6 | 0.1 | 3.8 | |
| Financial situation | No problem |  |  |  | | Ref |  |  | |  |  |  | |
|  | Tight or Difficult | **2.6** | **1.7** | **3.9** | | 1.2 | 0.6 | 2.3 | | 2.2 | 0.7 | 6.7 | |
| Same sex partnership | No |  |  |  | | Ref |  |  | |  |  |  | |
|  | Yes | 1.1 | 0.3 | 3.5 | | 1.9 | 0.9 | 4.0 | |  |  |  | |
|  | Never had sex | 0.1 | 0.0 | 0.8 | |  |  |  | |  |  |  | |
| Army rank | Enlist personnel |  |  |  | | Ref |  |  | |  |  |  | |
|  | Junior Officer | 0.7 | 0.5 | 1.0 | | 0.4 | 0.2 | 0.8 | | 0.8 | 0.4 | 1.8 | |
|  | Officer | 0.8 | 0.2 | 2.5 | | 0.1 | 0.0 | 0.6 | |  |  |  | |
| Deployment in the last 12 months | No |  |  |  | | Ref |  |  | |  |  |  | |
|  | Yes | 0.8 | 0.5 | 1.1 | | 0.4 | 0.2 | 0.8 | | 1.0 | 0.3 | 3.6 | |
| Military branch | Army |  |  |  | | Ref |  |  | |  |  |  | |
|  | Air force | 1.4 | 0.8 | 2.3 | | **3.2** | **1.7** | **6.1** | | 0.2 | 0.0 | 1.7 | |
|  | Navy | 0.9 | 0.6 | 1.1 | | 1.0 | 0.7 | 1.4 | | 0.1 | 0.0 | 0.4 | |
| Low acceptance of increasing female representation in the army | | 1.3 | 0.8 | 2.1 | | 0.8 | 0.5 | 1.3 | | 0.2 | 0.0 | 1.6 | |
| Low social cohesion |  | **0.9** | **0.8** | **1.0** | | 0.8 | 0.6 | 1.0 | | 0.8 | 0.5 | 1.5 | |
| Higher Female representation | | **0.7** | **0.6** | **0.9** | | 0.2 | 0.2 | 0.3 | | 1.1 | 0.5 | 2.2 | |

*sexual oppression includes sexual coercion, repeated verbal unwanted sexual attention, or sexual assault

Appendix 2. Odds of depressive symptoms and risks of positive PTSD score according to sexual stressors in the last 12 months among servicewomen in the French Military: results from multivariate and multinomial logistic regressions including unit level clustering.

|  |  | Depressive symptoms CESD score >10 vs CESD<=10 | | | Subtheshold PTSD vs No PTSD | | | | positive PTSD score vs No PTSD | | | |  |
| --- | --- | --- | --- | --- | --- | --- | --- | --- | --- | --- | --- | --- | --- |
|  |  | aOR | 95%CI | | | aRRR | 95%CI | | | aRRR | 95%CI | | |
|  | No MST | Ref |  |  | | Ref |  |  | | Ref |  |  | |
| MST | SH in the form of repeated sexual comments alone | **3.1** | **1.7** | **5.5** | | **4.5** | **2.8** | **7.4** | |  |  |  | |
|  | 1 form of sexual oppression* | **5.2** | **1.9** | **13.9** | | **3.9** | **1.6** | **9.6** | | **5.1** | **0.3** | **83.5** | |
|  | several sexual stressors | **6.5** | **2.6** | **16.0** | | **3.0** | **2.1** | **4.2** | | **11.3** | **2.3** | **55.6** | |
| Age | 18-24 | Ref |  |  | | Ref |  |  | | Ref |  |  | |
|  | 25-29 | 0.5 | 0.2 | 1.2 | | 0.5 | 0.2 | 1.7 | | 0.1 | 0.0 | 4.6 | |
|  | >=30 | **0.4** | **0.1** | **1.0** | | 1.4 | 0.5 | 3.9 | | 0.9 | 0.1 | 11.3 | |
| Cohabitation with current partner | Everyday | Ref |  |  | | Ref |  |  | | Ref |  |  | |
|  | Not every day | 2.2 | 0.7 | 7.0 | | 2.4 | 0.7 | 8.4 | | 1.4 | 0.3 | 6.4 | |
|  | No current partner | 2.3 | 0.7 | 8.0 | | 2.2 | 0.7 | 6.9 | | 1.5 | 0.1 | 37.8 | |
| Education level | <High school | Ref |  |  | | Ref |  |  | | Ref |  |  | |
|  | High school graduation | 0.8 | 0.3 | 2.3 | | 0.8 | 0.5 | 1.2 | | 2.2 | 0.2 | 7.2 | |
|  | >High school | 0.6 | 0.2 | 1.7 | | 1.3 | 0.4 | 4.2 | |  |  |  | |
| Financial situation | No problem | Ref |  |  | | Ref |  |  | | Ref |  |  | |
|  | Tight or Difficult | 1.3 | 0.5 | 3.6 | | 2.0 | 0.8 | 4.9 | | 7.3 | 0.9 | 59.5 | |
| Same sex partnership | No | Ref |  |  | | Ref |  |  | | Ref |  |  | |
|  | Yes | 1.1 | 0.6 | 1.9 | | 0.8 | 0.4 | 1.4 | | 0.7 | 0.2 | 1.8 | |
| Army rank | Enlist personnel | Ref |  |  | | Ref |  |  | | Ref |  |  | |
|  | Junior Officer | **2.2** | **1.0** | **5.0** | | 1.1 | 0.8 | 1.6 | | 0.4 | 0.6 | 2.8 | |
|  | Officer | 1.0 | 0.2 | 4.4 | | 0.5 | 0.1 | 2.2 | | 2.0 | 0.1 | 3.2 | |
| Deployment in the last 12 months | No | Ref |  |  | | Ref |  |  | | Ref |  |  | |
|  | Yes | 1.8 | 0.9 | 3.6 | | 0.8 | 0.3 | 2.2 | | 3.8 | 0.2 | 86.6 | |
| Military branch | Army | Ref |  |  | | Ref |  |  | | Ref |  |  | |
|  | Air force | **7.2** | **1.8** | **28.3** | |  |  |  | |  |  |  | |
|  | Navy | 1.5 | 0.9 | 2.4 | | 0.6 | 0.1 | 6.5 | |  |  |  | |
| Low acceptance of increasing female representation in the army | | 2.2 | 0.7 | 6.5 | | 4.8 | 0.8 | 28.9 | | 2.5 | 0.1 | 43.6 | |
| Low social cohesion | 0,8 | 0.8 | 0.5 | 1.2 | | 1.2 | 0.4 | 3.4 | | 1.1 | 0.1 | 17.7 | |
| Higher Female representation | | 1.1 | 0.5 | 2.0 | | 0.2 | 0.0 | 1.6 | | 0.1 | 0.0 | 2.2 | |

*sexual oppression includes sexual coercion, repeated verbal unwanted sexual attention, or sexual assault
